# An immersive virtual reality communication skills training for dietitians: A feasibility study

**DOI:** 10.1016/j.pecinn.2024.100292

**Published:** 2024-05-21

**Authors:** Ivan Moser, Victoria Mirata, Per Bergamin

**Affiliations:** a Institute for Research in Open, Distance, and eLearning, Swiss Distance University of Applied Sciences, Schinerstrasse 18, CH-3900 Brig, Switzerland; b UNESCO Chair on Personalised and Adaptive Distance Education, Swiss Distance University of Applied Sciences, Schinerstrasse 18, CH-3900 Brig, Switzerland; c Research Unit for Self-Directed Learning, North-West University of South Africa, Potchefstroom Campus, Private Bag X6001, Potchefstroom, SA 2520, South Africa

**Keywords:** Virtual reality, Dietetics, Communication skills training, Situated learning, Usability

## Abstract

**Objective:**

The training of dietitians emphasizes the development of strong communication skills. Immersive virtual reality (IVR) has been successfully employed for various healthcare scenarios; however, it is yet understudied in dietetics education. Therefore, we conducted a feasibility study to investigate the usefulness of IVR for the purpose of communication skills training.

**Methods:**

We designed a multi-user virtual hospital environment that enabled the training of professional conversations between dietitians and patients. Divided into groups of three, 30 dietetics students participated in a role-play task. Taking a qualitative approach to inquiry, we evaluated how participants assessed the benefits and limitations of the IVR training.

**Results:**

Participants appreciated the authenticity of the training environment and mentioned various advantages of IVR (e.g., sense of privacy, better focus on conversation) over traditional modes of instruction. On the other side, participants frequently mentioned that the lack of avatars' facial expressions might present an obstacle for effective communication skills training.

**Conclusion:**

IVR enables authentic communication skills trainings for dietitians. Special consideration should be given to providing ample social cues during training.

**Innovation:**

The study demonstrates that the promising results from other healthcare professions regarding the usefulness of IVR training also apply to dietetics.

## Highlights

• A virtual reality task was designed to train dietetic students' communication skills.
• Participants reported high authenticity of the virtual training.
• The lack of avatar facial expressions was perceived as a limitation.
• Virtual reality represents a promising tool to train dietitian-patient interactions.

## 1 Introduction

The growing prevalence and burden of nutritional diseases highlight the need for appropriate measures to address this global health issue [[1], [2], [3]]. Trained dietitians play an important role in the diagnosis and treatment of nutritional diseases. Being embedded in interdisciplinary teams, their profession is characterized by challenging and frequent conversations with patients and hospital staff (e.g., medical doctors, nurses, kitchen staff, etc.). Therefore, it is not surprising that the training of dietitians emphasizes the development of strong communication skills. Fostering communication skills is often achieved by including role-play exercises [4]. However, these training sessions can be costly and time-consuming, especially if they require the recruitment of professional actors.

Immersive virtual reality (IVR) enables the development of risk-free yet highly practice-oriented training, which cannot be delivered with other modes of instruction [5,6]. In this vein, classroom activities could be transferred to authentic learning environments, following the didactic principle of situated learning [[7], [8], [9]]. In short, situated learning theory states that the context in which knowledge or skills are acquired should closely resemble the context in which they are later applied. From the perspective of technology-enhanced learning, it has been argued that situated learning is one of the main mechanisms through which serious games support learning and skill acquisition [10,11]. In line with this notion, previous research has demonstrated the usefulness of IVR for the purpose of communication skills training [[12], [13], [14], [15]]. However, to our best knowledge, no applications have yet been tailored for the needs of dietitians.

Therefore, we set out to design and evaluate a multi-user IVR application that can be used for communication skills training by dietitians. Conducting a feasibility study, we investigated the usefulness of IVR in dietetics education by transferring an existing classroom activity to a virtual environment. We were interested in whether (1) the learning scenario would be perceived in line with the principles of situated learning (i.e., realism and authenticity of the hospital environment) and if (2) IVR would be perceived as a useful tool to promote communication skills.

## 2 Material and methods

### 2.1 Participants

We recruited 30 dietetics undergraduates at the Swiss Distance University of Applied Sciences (see Table 1 for demographic information). At the time of the study, all participants were enrolled in a course focusing on communication skills in a hospital context. Study participation was optional, and participants did not receive financial or other incentives for participation. All participants signed informed consent. All materials and procedures described below were approved by the Institutional Ethics Committee.

**Table 1:** Demographics and previous IVR experience of the sample.

N	Mean Age (SD)	Gender (f/m)	IVR experience (yes/no)
30	32.13 (7.57)	27 / 3	14 / 16

### 2.2 Material

The hospital environment and avatars were purchased on 3D model marketplaces and subsequently customized using a 3D modelling software (Maya 3D). The IVR application was programmed using the Unity game engine and deployed to Oculus Quest head-mounted displays (HMD's).

To evaluate the IVR application, we administered a feedback form with three exploratory, open-ended questions that addressed the perceived benefits and limitations of the IVR application [16]. In view of the limited prior research on the usefulness of IVR for dietetics education, this exploratory approach encouraged the participants to indicate any topics they deemed relevant for communication skills training with this technology. It allowed us to gather the relevant IVR design aspects that may inform the development of data collection instruments for later studies.

### 2.3 Procedure

The students signed up for the study in groups of three. At arrival, the participants completed a short demographics questionnaire. Afterwards, ten minutes were allocated for the participants to familiarize themselves with the IVR environment.

**Fig. 1:**
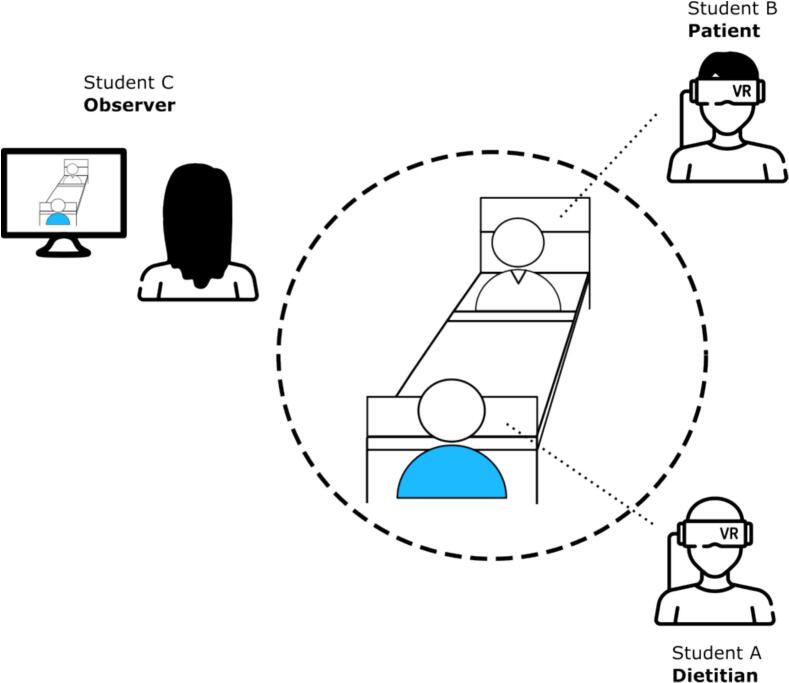
Schematic representation of the role-play task in the multi-user virtual environment. Adapted from Moser & Bergamin [17].

**Fig. 2:**
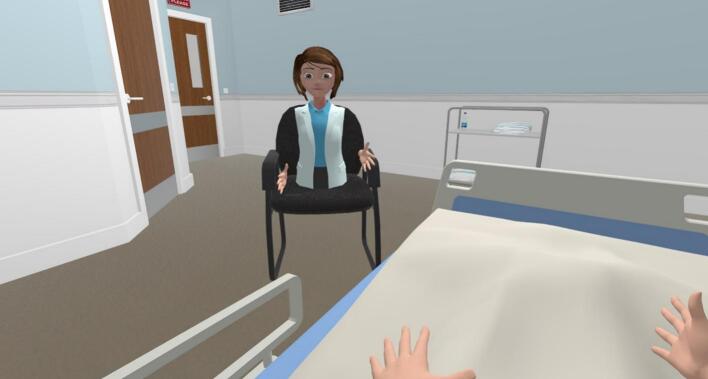
The virtual environment during the role play exercise as perceived from the perspective of the patient.

The three participants were then randomly assigned to the roles of the dietitian, patient, and observer. Then, they read the individual instructions for the role-play task. According to the task, the first student (A) impersonated a dietitian paying a visit to a hospitalized patient suffering from pneumonia and concomitant malnutrition, who was impersonated by student B. The third student (C) acted as an observer and followed the conversation from a desktop computer. A maximum of twenty minutes was allocated for the conversation, during which the participants communicated with each other in real-time, without the use of scripted questions or answers. Fig. 1 depicts a schematic representation of the roles in the role-play task. Fig. 2 provides a first-person view from the perspective of the patient role.

### 2.4 Analysis

**Fig. 3:**
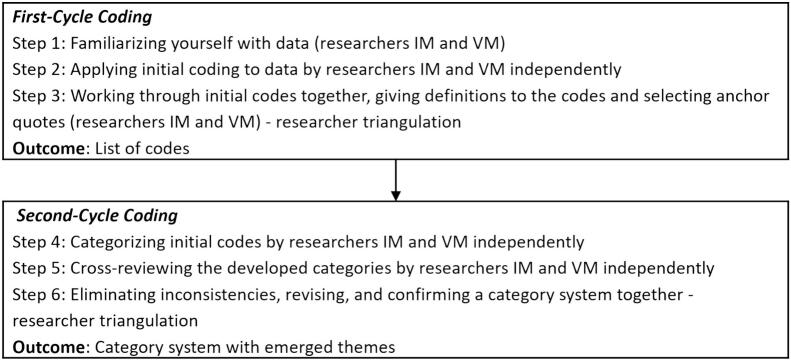
Data analysis procedure.

The students' answers were analyzed using inductive coding techniques (i.e., initial and thematic coding) conducted in multiple coding cycles [18]. The qualitative data were managed and analyzed in the qualitative data analysis software NVivo [19]. During the first coding cycle, the participants' responses were independently coded by two knowledgeable researchers. Afterwards, the researchers compared their initial codes and iteratively discussed them until they reached a consensus on the common code list. During the second coding cycle, the researchers jointly grouped the emerged codes into major categories and themes. The credibility of the data analysis was ensured through a careful documentation of all data analysis steps including definitions of theoretical concepts and researcher triangulation [20]. The detailed description of the data analysis procedure is displayed in Fig. 3.

## 3 Results

The qualitative data analysis of the perceived benefits yielded themes related to the general IVR experience, visualization of the hospital environment, ease of use, and advantages for counseling training. The themes for the perceived limitations included the visualization of avatars, ease of use, equipment and hardware, side effects, and insufficient functionality. Table 2 summarizes the emerged categories and themes, number of statements, and representative direct quotations, translated from German into English. The direct participants' quotes reflect the most typical responses of their roles (dietitian (D), patient (P), and observer (O)), identified during the data analysis process (i.e., anchor quotes). The reported benefits of IVR were consistent with situated learning principles such as realistic visualization of the hospital environment and the sense of being present in the virtual space. One participant noted, for example, that “the hospital atmosphere was more real compared to previous classroom exercises” (D5).^1^ Several participants commented positively on how they felt being in the virtual environment. One representative quotation was: “I felt like I was really lying as a patient in a hospital room, I even had the feeling of having a blanket over my legs” (P7). Only one participant in the role of observer found the hospital situation was “not real enough” (O1).

**Table 2:** Categories and themes of the benefits and limitations mentioned by the participants, including anchor quotes.

Benefits *“What did you like about the VR application?”*	f*	Anchor quotes
**General experience with IVR**		
Positive experience in general	9	“It was interesting to be in a virtual space. It certainly has potential.” (D7)
A new experience	3	“It's a new experience, something different, very interesting.” (O1)
A fun experience	3	“It was fun, great idea.” (P2)
A change from everyday life	2	“A change from everyday life” (P8)
**Ease of use**		
Easy and handy use	2	“Practical and simple” (P2)
Well-functioning technology	2	“It all worked well, once you knew how to use the functions and buttons.” (O10)
**Visualization of the hospital environment**		
Realistic visualization of the hospital environment (realness)	5	“The hospital atmosphere was more real compared to previous classroom exercises.” (D5)
Sense of being present in a virtual space (spatial presence)	5	“I felt like I was really in the patient room, so it all felt very real, and I could almost get lost in that situation. It is much closer to reality than when we do a triad exercise in an empty room.” (O10);
**Advantages for counseling training**		
Better empathy with the patient's situation	6	“You could get a real insight into the situation, it felt more real.” (P4).
Opportunity to practice patient counseling	4	“I think that a VR application cannot completely replace the face-to-face application, but it is a great tool for learning.” (D1)
Better focus on the conversation	2	“I felt more focused than usual, more serious, could put myself in the role better, less distracted.” (P10)
Sense of privacy due to IVR environment	1	“Since there are only two of you in the virtual room, you have the feeling of privacy (not being under observation).” (P7)
Sense of security due to IVR-glasses	1	“There was protection. When you practice counseling, you are also nervous, and the glasses gave me security.” (D10)
*Limitations* *“What did you dislike about the VR-application?”* *“Where do you see room for improvement?”*		
**Visualization of avatars**		
Lack of facial expressions, gestures	11	“The interpersonal aspects such as emotions of posture and facial expression are missing. You are less able to interpret the person in front of you and respond to him/her personally.” (O5)
Incomplete avatars (e.g., no hands)	3	“The entire person was not visible.” (O6)
Unrealistic visualization of avatars	1	“Quite real and yet not completely real. My partner looked like a character in an animated film and not like a real person. I found that a bit disturbing for practicing the counseling setting.” (O2)
**Visualization of the hospital environment**		
Unrealistic visualization of the hospital setting	1	“Hospital situation is not real enough.” (O1)
Distracting elements or bugs (e.g., hopping bottle)	1	“A bottle in the background [in the virtual environment] bounced up and down all the time.” (O1)
**Ease of use**		
Needs to get used to	2	“On the one hand it took a bit of getting used to, but I found it exciting to be able to get to know [the VR application] this way.” (P6)
A little complicated	1	“A bit complicated” (P1)
**Equipment / Hardware**		
Uncomfortable VR-glasses	6	“The glasses are very heavy and, together with the face mask, not very comfortable to wear.” (D2); “Glasses become very heavy over time, image quality becomes tiring.” (D9); “Pressure on the nose” (D6)
Insufficient sound quality (e.g., low volume, delay)	6	“Sound is very quiet, so you have to speak very loudly.” (P5); “I understood the patient very poorly and had to speak very loudly.” (D5)
Blurred vision	6	“The head support was not tight enough on me (couldn't be retightened), so my vision was a bit blurry, and I had to hold the glasses a bit sometimes as a patient to have a sharp vision.” (P6); “Sometimes slightly blurry vision. I think you have to sit, when you use [the VR application], otherwise it's annoying.” (D10)
**Side effects**		
Dizziness and Nausea (Cybersickness)	2	“I felt a bit nauseous during the application, a bit dizzy after taking off the glasses.” (P10)
Distorted perception of space and time	2	“You lose the sense of time and place.” (P5)
**Insufficient functionality of the IVR application**		
Lacking possibility to take notes during the conversation	1	“There are no possibilities to take notes during the conversation or to take notes with you.” (P8)
Unavailability of nutrition reports	1	“Reading the case study virtually was a bit more difficult than in the real environment. Besides, the most important information for me (nutrition protocol) was not visible.” (D1)4

*Note.* * f = number of statements.

Several participants mentioned benefits regarding the possibility to practice counseling in a hospital room. They reported that using IVR for training helped them to familiarize themselves with the future counseling situation, to stay more focused on the conversation, and to experience a sense of privacy and security. Two representative quotations were: “You could get a real insight into the situation, it felt more real” (P4); “There was protection. When you practice counseling, you are also nervous, and the glasses gave me security” (D10).

The most frequently mentioned limitation of the IVR application was linked to the visualization of avatars, namely the lack of facial expressions and gestures, which were deemed crucial for counseling: “Facial expressions and gestures are missing, body language is a very important point in counseling, this is unfortunately missing here” (P4); “Emotions of the people involved can only be judged by the pitch of their voices, as gestures and facial expressions are completely absent. I find this crucial for a counseling situation” (O1). Other limitations were linked to equipment and hardware like uncomfortable HMDs, blurred vision, and insufficient sound quality. Moreover, five people mentioned slight discomfort (dizziness and nausea, distorted perception of space and time, etc.) and two people mentioned that the application was missing certain functionality (e.g., note taking).

## 4 Discussion and conclusion

### 4.1 Discussion

We found evidence that IVR allows to create purposeful communication skill trainings for dietetic students. This is in line with the perception of domain experts, who attested to IVR's high potential for the training of healthcare professionals in general [21]. Furthermore, we were able to design an IVR experience that was perceived by many as highly realistic and beneficial compared to classroom-based trainings. Both indicates that the IVR application succeeded to serve as a situated learning experience. This is important, because it has repeatedly been put forward that situatedness is a driving force for skill acquisition in IVR [10,11].

At the same time, our results pointed out important caveats. Many participants considered the lack of facial expressions as an obstacle for social skills training. Again, their criticism is aligned with domain experts, who characterized the avatars of existing IVR applications in the healthcare domain as not being realistic, which may limit the ability of healthcare professionals to immerse themselves in a training scenario [21].

It is noteworthy, however, that several participants reported that they felt more immersed in the situation compared to classroom-based trainings. In fact, the cognitive benefits (e.g., being more focused) might overrule the limits imposed by the lack of facial expressions. The lack of facial expression could even be regarded as an asset rather than a limitation if it enables social skills trainings that focus on paraverbal communication (e.g., pitch, tone, speed etc.).

### 4.2 Innovation

Our study investigated IVR training methods that have already been implemented successfully in other fields (e.g. nursing and medical training) and applied them to dietetics education. It corroborates the notion that IVR represents a viable alternative to traditional communication skills training in the classroom and that this promising finding extends from other healthcare professions to dietitians.

### 4.3 Conclusion

IVR provides an authentic, situated learning experience for future dietitians. For successful implementation, however, special attention should be given to providing ample social cues, which are considered critical for successful training.

## Funding

This work was supported by the 10.13039/501100001711Swiss National Science Foundation [grant number CRSK-1_190636 / 1].

## CRediT authorship contribution statement

**Ivan Moser:** Writing – review & editing, Writing – original draft, Visualization, Software, Resources, Methodology, Investigation, Funding acquisition, Formal analysis, Conceptualization. **Victoria Mirata:** Writing – review & editing, Writing – original draft, Methodology, Formal analysis. **Per Bergamin:** Writing – review & editing, Supervision.

## Declaration of competing interest

The authors declare that they have no known competing financial interests or personal relationships that could have appeared to influence the work reported in this paper.

## Footnotes

1 The letters in the brackets represents the participant code used for anonymization. The first letter of the code stands for the role during the conversation (D = dietitian, P = patient, O = observer).
